# The GG genotype of rs743572 in CYP17A1 gene regulating the decrease of T/E ratio can be an independent risk factor for MetS-BPH: a retrospective cohort study

**DOI:** 10.1007/s00345-024-05138-3

**Published:** 2024-07-24

**Authors:** Congcong Chen, Ningrui Pan, Zongping Chen, Chengren Gou, Xu He, Min Wang, Bo Chen, Zidong Zhou, Qixu Ren, Youzhuang Zhong, You Xiang, Sicong Zhao, Yong Yan, Tao Song

**Affiliations:** 1 Department of Urology, Affiliated Hospital of Zunyi Medical University, Zunyi, Guizhou 563000 China; 2 Department of Urology, People’s Hospital of Wuchuan County, Wuchuan, Guizhou 564300 China; 3 Department of Urology, Beijing Shijitan Hospital, Capital Medical University, Beijing, 100038 China; 4 Department of Urology, Eastern Hospital, Sichuan Academy of Medical Sciences & Sichuan Provincial People’s Hospital, Chengdu, 610101 China

**Keywords:** Metabolism syndrome, Benign prostate hyperplasia, Sex hormones, Gene polymorphism, Single nucleotide polymorphisms

## Abstract

**Purpose:**

To confirm if the CYP17A1 gene regulates the ratio of T/E leading to MetS-BPH.

**Methods:**

824 men, aged 47–88 years, were recruited into this study through consecutive routine physical examination programs and long-term outpatient screening. Several parameters, including SNPs of CYP17A1 gene, total testosterone, estradiol, and the ratio of total testosterone to estradiol (T/E) were obtained for each participant. Based on the diagnosis of BPH, MetS, and MetS-BPH, the participants were divided into BPH and non-BPH groups, MetS and non-MetS groups, and MetS-BPH and non-MetS-BPH groups. Values of the obtained parameters were evaluated using one-way analysis of variance, Student’s t-test, Chi-squared test, and logistic regression analysis.

**Results:**

SNPs of the CYP17A1 gene, including the rs743572 genotypes (GG, GA, and AA), rs3781287 genotypes (GG, GT, TT), and rs4919686 genotypes (CC, CA, and AA), were present in every group. Only the GG genotype of rs743572 was independently associated with BPH (OR = 5.868, 95% CI: 3.363–7.974, *P* < 0.001), MetS (OR = 7.228, 95% CI: 3.925–11.331, *P* < 0.001), and MetS-BPH (OR = 3.417, 95% CI: 1.783–5.266, *P* < 0.001) after adjusting for age. In the population of genotype GG of rs743572, the decrease in T/E ratio was an independent risk factor for BPH (OR = 839.756, 95% CI: 36.978-1334.263, *P* = 0.001), MetS (OR = 376.988, 95% CI: 12.980-488.976, *P* < 0.003), and MetS-BPH (OR = 388.236, 95% CI: 24.869-495.363, *P* = 0.003).

**Conclusion:**

The GG genotype of rs743572 in CYP17A1 gene regulating the decrease of T/E ratio can be an independent risk factor for MetS-BPH populations.

**Trial registration number:**

ChiCTR2200057632 “retrospectively registered”.

**Date of registration:**

March 15, 2022 “retrospectively registered”.

**Supplementary Information:**

The online version contains supplementary material available at 10.1007/s00345-024-05138-3.

## Introduction

Metabolic syndrome (MetS) is a complex medical disorder associated with a constellation of metabolic abnormalities. According to the most widely accepted definition, patients with at least 3 of the following risk factors are considered to have MetS: abdominal obesity, hypertriglyceridemia, low high-density lipoprotein cholesterol, high blood pressure, and high fasting blood glucose levels [1]. With the improvement of living standards, the incidence of MetS has risen sharply, becoming a serious public health problem in modern society [2, 3]. It is estimated that the incidence of MetS in China is more than 25%, with more than 100 million patients [4, 5].

Benign prostatic hyperplasia (BPH) is a histologic diagnosis that refers to the proliferation of glandular epithelial tissue, smooth muscle, and connective tissue within the prostatic transition zone [6, 7]. BPH is likely a multifactorial process, the exact etiology of which is unknown, but requires testosterone. BPH can lead to benign prostatic enlargement (BPE), which can obstruct at the level of the bladder neck, termed benign prostatic obstruction (BPO), and the prevalence and severity of lower urinary tract symptoms (LUTS) in aging men are progressive and impact the health and welfare of society [6, 7]. Studies have shown that 48.59% of BPH are combined with MetS (MetS-BPH) and there is a high degree of correlation between their pathogenesis, occurrence, and development [8–16]. The imbalance of sex hormones is an important cause of BPH and the main reason for the emergence of insulin resistance (IR) in MetS. Testosterone replacement therapy (TRT) helps to prevent and improve MetS. It has been shown that TRT can improve LUTS, improve international prostate symptom scores (IPSS), decrease residual urine, and increase the maximum urinary flow rate in BPH. However, it does not increase the volume of the prostate and PSA levels [14–16]. This indicates that a decrease in androgens may be involved in the occurrence of MetS-BPH.

Although the genetics of MetS-BPH are still not fully understood, published studies suggest that sex hormone metabolism-related gene polymorphisms may be involved in the occurrence of MetS-BPH. Although no high penetrance genetic markers for BPH and MetS have been identified yet, certain alleles of genes have been associated with diseases [17–20]. Cytochrome P450c17α (CYP17A1) gene, found in the 10q24.32 chromosomal region and encoding cytochrome P450c17α protein, is an enzyme with 17α-hydroxylase and 17, 20-lyase activities at branch points in estrogen and testosterone biosynthesis [19, 20]. It can regulate sex hormones in the body and maintain the dynamic balance of estrogen and androgens. Because the existence of the gene polymorphism may cause changes in the ratio of male testosterone and estrogen at different age stages, the occurrence of MetS-BPH often results [15, 16]. This study aimed to confirm if the CYP17A1 gene regulates the ratio of T/E leading to MetS-BPH.

## Subjects/Materials and methods

### Patients

This study was a retrospective cohort study. From October 2014 to March 2022, 824, community elderly male residents who had an IPSS evaluation and had consecutively participated in prostate examinations at Beijing Shijitan Hospital and Affiliated Hospital of Zunyi Medical University were recruited into this study. To minimize potential confounding factors and bias, the participants who had a history of prostate or urethral surgery; those who had been diagnosed with urologic diseases, including urethral stricture, urologic infections, malignancy, or neurogenic bladder; and those who had been administrated drugs, including anticholinergics, 5a-reductase inhibitors, phosphodiesterase-5 inhibitors, and hormone replacement therapy were excluded from the study.

### Blood specimen collection, detection, and cryopreservation

All blood specimens were obtained. Serum prostate-specific antigen (PSA) levels, serum total testosterone (TT), estradiol (E2), dihydrotestosterone (DHT), insulin (INS), androgen binding globulin (SHBG), fasting plasma glucose (FPG), triglycerides, HDL-C, and total cholesterol (TC) were determined. The insulin resistance index (HOMA-IR) was calculated and genomic DNA was extracted. The T/E ratio was calculated using serum total testosterone divided by estradiol.

### Single nucleotide polymorphism (SNP) selection and genotyping

The human genome database NCBI was used to search the human CYP17A1 gene and determine its study region. The human genome haplotype SNP database was used to search the SNP genotyping data in the study region, and the genotyping data of CYP17A1 SNPs in the Chinese Han population were obtained. The obtained genotyping data were imported into the Sequenom software package, the SNPs with minimum allele frequency greater than 0.20 were screened, and the linkage disequilibrium map and data were derived. According to the haplotype region construction criteria, SNPs with *R*^2^ (Coefficient of determination) greater than 0.8 were selected as the selected SNP sites. Eventually, three SNPs were selected, including rs743572 located in the 5’-untranslated (5’-UTR) promoter region of CM000672.2 (102837395A > G), rs3781287 located in the intron region of NM_000102.3 (298-271A > C), and rs4919686 located in the intron region of NM_000102.3 (1139 + 19T > G) of the CYP17A1 gene (http://www.ncbi.nlm.nih.gov/SNP), for analysis. PCR amplified target sequences included rs742572 (5’-CACAGCTCTTCTACTCCACC-3’ and 5’-CACAGCTCTTCTACTCCACT-3’, 130 base pairs), rs3781287 (5’-CAGCATTGGGGTTCTG-3’ and 5’-CAGCATTGGGGTTCTT-3’, 125 base pairs), and rs4919686 (5’-CCCCTGTGCCTGCCCTCCCAGG-3’ and 5’-CCCCTGTGCCTGCCCTCCCAGT-3’, 143 base pairs). Matrix-assisted laser resolution ionization flight time mass spectrometry was used for detection. Typer 4.0 software was used to detect the mass spectra and the genotypes of each sample target site were read according to the mass spectra. Assay design, DNA isolation, PCR amplification, direct sequencing, and analysis were performed with the iPLEX MassARRAY platform (Sequenom) [21, 22, 23]

### Assessment and definition of BPH

The subjects’ medical histories were collected using a standardized structured questionnaire. The Chinese version of the IPSS was administered to the subjects to evaluate urinary symptoms. Prostate volume (PV) was measured using transrectal ultrasonography, and the PV was calculated using the prolate ellipse formula (transverse × anteroposterior × cephalocaudal diameter × π/6). The maximum urinary flow rate (Qmax) was determined by uroflowmetry at a voided volume of > 150 mL [15, 16]. All subjects underwent digital examinations of the rectum to exclude palpable prostatic nodules. According to the results from the placebo-arm study of the Medical Therapy of Prostatic Symptoms study (MTOPS), TPV > 20 ml was defined as prostatomegaly (BPE), and the defined predictors for clinical BPH progression included a TPV ≥ 31 ml, Qmax < 10.6 ml/s, PSA ≥ 1.6 ng/ml, and age of 45 years or older [24]. The reference value of PSA in BPH should meet the following requirements: (i) PSA less than 4ng/ml; (ii) When PSA is in the gray zone range of 4-10ng/ml, the ratio of free PSA to total PSA should be greater than 0.16, PSA density (PSAD) should be less than 0.15, PSA velocity (PSAV) should be less than 0.75ng/ml.y, otherwise prostate biopsy should be performed to exclude prostate cancer; (iii) When PSA was more than 10ng/ml, prostate biopsy was required to exclude prostate cancer before enrollment in this study [6, 7].

### Definition of MetS

MetS was diagnosed using the 2005 National Cholesterol Education Program-Adult Treatment Panel III (NCEP-ATP III) criteria for Asian Americans [1]. Specific diagnostic criteria are detailed in reference 15.

### Definition of MetS-BPH and Normal

MetS-BPH refers to the above diagnostic criteria of MetS and BPH, and the occurrence of MetS simultaneously with BPH. Normal refers to the indicators in this study that do not have diagnostic MetS, BPH, and other diseases, and the physical examination indicators are normal, and meet the Qmax > 20 ml/s.

### Data extraction

The following parameters were obtained from each participant: age (year), TT, E2, DHT, INS, FPG, SHBG, IPSS, Qmax, PSA, TPV, HOMA-IR, SNPs (rs743572, rs3781287, rs4919686) of CYP17A1.

### Statistical analyses

Statistical analyses were performed using Statistical Package for the Social Sciences (IBM Corp., Armonk, NY, USA) version 29.0. The selected characteristics were expressed as mean and standard deviation (mean ± SD) as well as percentage (%) for comparison between BPH and Non-BPH groups, MetS and Non-MetS groups, as well as MetS-BPH and Non-MetS-BPH groups. Student’s t-test and one-way analysis of variance (one-way ANOVA) were used for continuous variables; allelic and genotypic associations were evaluated by chi-square test; multivariate-adjusted odds ratios (ORs) and 95% confidence intervals (CIs) were simultaneously estimated by logistic regression analyses. Differences are considered statistically significant when *P* < 0.05.

## Results

### Patient characteristics

The principal characteristics of the study population are listed in Table 1. A total of 824 men (47–88 years) were analyzed. These parameters, including age, TT, E2, T/E, INS, FPG, SHBG, DHT, IPSS, Qmax, PSA, TPV, and HOMA-IR, in the BPH group, MetS group, MetS-BPH group, and Normal group, respectively, were compared, except DHT (*P* = 0.969), differences were statistically significant (all *P* < 0.05).

**Table 1:** Principal cohort characteristics (*n* = 824)

Variables	BPH (*n* = 476)	MetS (*n* = 52)	MetS-BPH (*n* = 177 )	Normal (*n* = 119)	*P*-value
Age, years	70.80 ± 7.95	64.65 ± 9.23	68.67 ± 7.97	68.00 ± 8.03	**< 0.001**
TT, ng/ml	4.94 ± 1.35	4.04 ± 1.07	3.93 ± 1.26	5.15 ± 1.48	**< 0.001**
E2, pg/ml	44.59 ± 17.59	33.48 ± 13.42	43.04 ± 19.52	31.01 ± 7.20	**< 0.001**
T/E, ng/pg	0.13 ± 0.06	0.14 ± 0.05	0.11 ± 0.07	0.18 ± 0.08	**< 0.001**
INS, µIU/ml	46.15 ± 26.29	64.23 ± 32.78	58.02 ± 21.12	52.44 ± 36.31	**0.003**
FPG, mmol/l	5.45 ± 1.24	6.78 ± 1.31	6.71 ± 1.24	5.23 ± 1.28	**0.006**
SHBG, nmol/l	75.81 ± 35.91	51.36 ± 23.20	60.17 ± 25.67	73.72 ± 39.28	**< 0.001**
DHT, pg/ml	402.35 ± 128.59	369.09 ± 166.20	393.68 ± 164.86	395.12 ± 132.37	0.969
IPSS	17.73 ± 5.70	3.10 ± 2.47	18.16 ± 5.59	3.06 ± 2.51	**< 0.001**
Qmax, ml/s	13.60 ± 3.30	20.50 ± 2.20	11.66 ± 2.91	22.48 ± 1.54	**< 0.001**
PSA, ng/ml	1.58 ± 1.53	1.32 ± 0.73	2.32 ± 1.44	1.14 ± 0.72	**< 0.001**
TPV, ml	28.16 ± 7.01	17.59 ± 1.79	28.66 ± 6.86	17.54 ± 1.86	**< 0.001**
HOMA-IR	1.49 ± 0.23	2.86 ± 1.54	2.38 ± 1.09	1.64 ± 1.02	**0.002**

*Abbreviations* TT, total testosterone; E2, estradiol; T/E, total testosterone/estradiol; DHT, dihydrotestosterone; INS, insulin; FPG, fasting plasma glucose; SHBG, sex hormone binding globulin; IPSS, international prostate symptom score; Qmax, maximum urinary flow rate; PSA, prostate specific antigen; TPV, total prostate volume; HOMA-IR, insulin resistance index; BPH, benign prostatic hyperplasia; MetS, metabolic syndrome

*P*-values were calculated using one-way analysis of variance (one-way ANOVA), and Bonferroni’s correction be used

The boldface represents statistical significance (*P* < 0.05)

### Genotypes and allele distributions of rs743572, rs3781287 and rs4919686 in BPH and non-BPH groups, as well as a comparison between the two groups

The genotypic distribution of rs743572, rs3781287, and rs4919686 in study subjects was in accordance with Hard-Weinberg equilibrium (data not shown). As shown in Table 2, the genotypes GG, GA and AA of rs743572 had statistically significant differences between BPH and non-BPH groups (*P* < 0.001). However, only the genotypes GG (OR = 5.868, 95% CI: 3.363–7.974, *P* < 0.001) of rs743572 were independently associated with BPH after adjusting for age, TT, E2, T/E, INS, FPG, SHBG, DHT, IPSS, Qmax, PSA, TPV, and HOMA-IR. Both the genotypes GG, GT and TT of rs3781287 (*P* = 0.958) and the genotypes CC, CA and AA of rs4919686 (*P* = 0.071) had not statistically significant differences between BPH and non-BPH groups.

**Table 2:** Genotypes and allele distributions of rs743572, rs3781287, and rs4919686 in BPH and non-BPH groups and a comparison between the two groups (*n* = 824)

SNP	Type	BPH, Ratio (%)	Non-BPH, Ratio (%)	*P*-value^1^	Model	OR (95% CI)	*P*-value^2^
rs743572	GG	301/653 (46.09)	44/171 (25.73)	**< 0.001**		5.868 (3.363–7.974)	**< 0.001**
	GA	178/653 (27.26)	62/171 (36.26)			.	.
	AA	174/653 (26.65)	65/171 (38.01)		Allele	1.102 (1.021–2.243)	0.059
rs3781287	GG	211/653 (32.31)	52/171 (30.41)	0.958		.	.
	GT	291/653 (44.56)	77/171 (45.03)			.	.
	TT	151/653 (23.13)	42/171 (24.56)		Allele	.	.
rs4919686	CC	12/653 (1.84)	7/171 (4.01)	0.071		0.495 (0.102–2.399)	0.382
	CA	130/653 (19.91)	40/171 (23.39)			.	.
	AA	511/653 (78.25)	124/171 (72.60)		Allele	1.420 (0.792–2.545)	0.239

*Abbreviation* BPH, benign prostatic hyperplasia; SNP, single nucleotide polymorphisms; OR, odds ratio; CI, confidence interval

^1^Chi-squared test

^2^Multivariate logistic regression analysis, adjusted for age, TT, E2, T/E, INS, FPG, SHBG, DHT, IPSS, Qmax, PSA, TPV, and HOMA-IR

The boldface represents statistical significance (*P* < 0.05)

### Genotypes and allele distributions of rs743572, rs3781287, and rs4919686 in MetS and non-MetS group, as well as a comparison between the two groups

As shown in Table 3, both the genotypes GG, GA and AA of rs743572 (*P* = 0.008) and the genotypes GG, GT and TT of rs3781287 (*P* = 0.014) had statistically significant differences between MetS and non-MetS groups. However, only the genotype GG of rs743572 was independently associated with MetS (OR = 7.228, 95% CI: 3.925–11.331, *P* < 0.001) after adjusting for age, TT, E2, T/E, INS, FPG, SHBG, DHT, IPSS, Qmax, PSA, TPV, and HOMA-IR. The genotypes CC, CA and AA of rs4919686 had not statistically significant differences between MetS and non-MetS groups (*P* = 0.767).

**Table 3:** Genotypes and allele distributions of rs743572, rs3781287, and rs4919686 in MetS and non-MetS groups and a comparison between the two groups (*n* = 824)

SNP	Type	MetS, Ratio (%)	Non-MetS, Ratio (%)	*P*-value^1^	Model	OR (95% CI)	*P*-value^2^
rs743572	GG	109/229 (47.60)	236/595 (39.66)	**0.008**		7.228 (3.925–11.331)	**< 0.001**
	GA	73/229 (31.88)	225/595 (37.82)			.	.
	AA	47/229 (20.52)	134/595 (22.52)		Allele	1.224 (0.322–2.143)	0.836
rs3781287	GG	78/229 (34.06)	185/595(31.09)	**0.014**		.	.
	GT	92/229 (40.17)	261/595(43.87)			.	.
	TT	59/229(25.77)	149/595 (25.04)		Allele	1.248 (0.848–2.267)	0.887
rs4919686	CC	5/229 (2.18)	14/595 (2.35)	0.767		0.728 (0.127–4.163)	0.721
	CA	44/229 (19.21)	111/595 (18.66)			.	.
	AA	180/229(78.61)	470/595(78.99)		Allele	1.013 (0.432–2.124)	0.824

*Abbreviation* MetS, metabolic syndrome; SNP, single nucleotide polymorphisms; OR, odds ratio; CI, confidence interval

^1^Chi-squared test

^2^Multivariate logistic regression analysis, adjusted for age, TT, E2, T/E, INS, FPG, SHBG, DHT, IPSS, Qmax, PSA, TPV, and HOMA-IR

The boldface represents statistical significance (*P* < 0.05)

### Genotypes and allele distributions of rs743572, rs3781287, and rs4919686 in MetS-BPH and non-MetS-BPH group, as well as a comparison between the two groups

As shown in Table 4, the genotypes GG, GA and AA of rs743572 had statistically significant differences between MetS-BPH and non-MetS-BPH groups (*P* < 0.001). However, only the genotype GG of rs743572 was independently associated with MetS-BPH (OR = 3.417, 95% CI: 1.783–5.266, *P* < 0.001) after adjusting for age, TT, E2, T/E, INS, FPG, SHBG, DHT, IPSS, Qmax, PSA, TPV, and HOMA-IR. Both the genotypes GG, GT and TT of rs3781287 (*P* = 0.089) and the genotypes CC, CA and AA of rs4919686 (*P* = 0.475) had not statistically significant differences between MetS-BPH and non-MetS-BPH groups.

**Table 4:** Genotypes and allele distributions of rs743572, rs3781287, and rs4919686 in MetS-BPH and non-MetS-BPH groups and a comparison between the two groups (*n* = 824)

SNP	Type	MetS-BPH, Ratio (%)	Non-MetS-BPH, Ratio (%)	*P*-value^1^	Model	OR (95% CI)	*P*-value^2^
rs743572	GG	96/177 (50.28)	249/647 (38.49)	**< 0.001**		3.417 (1.783–5.266)	**< 0.001**
	GA	50/177 (32.20)	229/647(35.39)			.	.
	AA	31/177(17.51)	169/647 (26.12)		Allele	1.425 (1.302–2.723)	0.927
rs3781287	GG	61/177 (34.46)	202/647 (31.22)	0.089		.	.
	GT	70/177 (39.55)	299/647 (46.21)			.	.
	TT	46/177 (25.99)	146/647 (22.57)		Allele	2.012(1.636–3.894)	0.835
rs4919686	CC	4/177 (2.26)	16/647 (2.47)	0.475			
	CA	36/177 (20.34)	134/647 (20.71)			.	.
	AA	137/177 (77.40)	497/647 (76.82)		Allele	.	.

*Abbreviation* BPH, benign prostatic hyperplasia; MetS, metabolic syndrome; MetS-BPH, BPH combined with MetS; SNP, single nucleotide polymorphisms; OR, odds ratio; CI, confidence interval

^1^Chi-squared test

^2^Multivariate logistic regression analysis, adjusted for age, TT, E2, T/E, INS, FPG, SHBG, DHT, IPSS, Qmax, PSA, TPV, and HOMA-IR

The boldface represents statistical significance (*P* < 0.05)

### Association between diseases of BPH, MetS, and MetS-BPH and related parameters of sex hormones, BPH, and MetS in the genotype GG of rs743572

As shown in Table 5, this study further analyzed the relationship between genotype GG of rs743572 and all related parameters of sex hormones, BPH, and MetS, and only T/E had a common independent association with the genotype GG of rs743572 in the BPH group (OR = 839.756, 95% CI: 36.978-1334.263, *P* = 0.001), MetS group (OR = 376.988, 95% CI: 12.980-488.976, *P* = 0.003), and MetS-BPH group (OR = 388.236, 95% CI: 24.869-495.363, *P* = 0.003).

**Table 5:** Association between GG-genotype of rs743572 and clinical related parameters of sex hormones, BPH, and MetS.

Parameters	rs743572 type GG (*n* = 345)															
	BPH					MetS					MetS-BPH					
	value	*P*-value^3^	OR	*P*-value^4^	95%CI	value	*P*-value^3^	OR	*P*-value^4^	95%CI	value	*P*-value^3^	OR	*P*-value^4^	95%CI	
Age, years																
(+)^1^	69.76 ± 7.84	**0.022**	1.131	0.198	0.973–1.181	66.91 ± 8.40	0.067	1.106	0.325	0.905–1.353	68.72 ± 7.32	0.334	0.998	0.959	0.938–1.063	
(-)^2^	66.28 ± 8.73					67.72 ± 7.72					69.24 ± 9.04					
TT, ng/ml																
(+)^1^	4.72 ± 1.71	0.178	1.005	0.976	0.731–1.384	3.89 ± 1.28	**0.004**	0.308	0.113	0.072–1.323	4.06 ± 1.26	0.074	0.645	0.073	0.421–1.047	
(-)^2^	4.52 ± 1.43					4.73 ± 1.73					4.59 ± 1.71					
E2, pg/ml																
(+)^1^	43.56 ± 15.28	**0.026**	0.981	0.513	0.945–1.038	38.24 ± 11.54	0.344	1.084	0.132	0.989–1.199	42.62 ± 13.83	**0.023**	1.024	0.792	0.971–1.048	
(-)^2^	33.42 ± 11.83					37.52 ± 12.60					38.52 ± 14.67					
T/E, ng/pg																
(+)^1^	0.13 ± 0.04	**0.010**	839.756	**0.001**	36.978-1334.263	0.13 ± 0.09	**0.032**	376.988	**0.003**	12.980-488.976	0.11 ± 0.08	**0.003**	388.236	**0.003**	24.869-495.363	
(-)^2^	0.16 ± 0.10					0.15 ± 0.09					0.15 ± 0.07					
INS, µIU/ml																
(+)^1^	48.58 ± 27.16	0.054	1.053	0.356	0.924–1.126	59.89 ± 30.11	0.055	1.449	**0.028**	1.184–1.941	58.10 ± 19.37	0.627	1.193	0.061	0.996–1.498	
(-)^2^	58.28 ± 27.78					52.68 ± 34.25					56.46 ± 32.06					
FPG, mmol/l																
(+)^1^	5.49 ± 1.04	0.657	1.468	0.293	0.759–2.264	6.86 ± 1.49	**< 0.001**	50.817	0.101	0.464–57.353	6.77 ± 1.15	**< 0.001**	8.988	**< 0.001**	3.091–32.445	
(-)^2^	5.67 ± 1.66					5.01 ± 0.48					5.25 ± 1.04					
SHBG, nmol/l																
(+)^1^	75.56 ± 34.49	**0.028**	1.032	0.746	0.948–1.147	57.96 ± 24.87	0.076	1.046	0.358	0.971–1.184	60.53 ± 26.24	**0.036**	1.024	0.143	0.953–1.065	
(-)^2^	63.81 ± 35.42					73.42 ± 36.98					68.56 ± 36.48					
DHT, pg/ml																
(+)^1^	395.42 ± 129.06	0.385	1	0.523	0.999–1.002	384.96 ± 144.71	0.726	1	0.945	0.993–1.017	393.33 ± 189.61	0.989	1	0.387	1.000-1.003	
(-)^2^	388.99 ± 216.29					396.15 ± 208.12					392.48 ± 171.44					
IPSS																
(+)^1^	17.98 ± 4.58	**< 0.001**	3.421	**< 0.001**	1.128–4.682	13.77 ± 8.68	0.482	1.012	0.685	0.954–1.074	18.17 ± 5.88	**< 0.001**	1.338	0.188	0.967–1.526	
(-)^2^	3.02 ± 2.49					12.72 ± 7.22					11.24 ± 7.55					
Qmax, ml/s																
(+)^1^	12.63 ± 2.82	**< 0.001**	4.134	**0.010**	3.030–5.249	14.50 ± 5.17	**0.002**	0.900	0.067	0.804–1.007	11.02 ± 2.26	**< 0.001**	0.976	0.587	0.842–1.188	
(-)^2^	22.20 ± 2.65					17.55 ± 6.06					17.35 ± 6.00					
PSA, ng/ml																
(+)^1^	1.58 ± 1.57	0.112	1.175	0.313	0.852–1.675	1.38 ± 0.70	0.067	0.656	0.462	0.212–1.026	1.67 ± 0.75	0.087	0.845	0.493	0.514–1.364	
(-)^2^	1.16 ± 1.02					1.22 ± 1.55					1.33 ± 1.29					
TPV, ml																
(+)^1^	28.24 ± 6.13	**< 0.001**	3.256	0.056	0.980–3.837	25.53 ± 7.64	0.406	1.002	0.967	0.917–1.094	28.69 ± 6.51	**0.002**	0.976	0.794	0.881–1.141	
(-)^2^	17.16 ± 2.73					25.38 ± 7.64					21.64 ± 7.55					
HOMA-IR																
(+)^1^	1.57 ± 0.97	**0.035**	1.324	0.227	0.413–2.178	2.57 ± 1.34	**< 0.001**	2.326	**< 0.001**	1.357–4.126	2.64 ± 0.75	**< 0.001**	3.886	**< 0.001**	2.916–4.595	
(-)^2^	2.29 ± 1.89					1.58 ± 1.23					1.51 ± 1.43					

*Abbreviations* BPH, benign prostatic hyperplasia; TT, total testosterone; E2, estradiol; T/E, total testosterone/estradiol; DHT, dihydrotestosterone; INS, insulin; FPG, fasting plasma glucose; SHBG, sex hormone binding globulin; IPSS, international prostate symptom score; Qmax, maximum urinary flow rate; PSA, prostate specific antigen; TPV, total prostate volume; HOMA-IR, insulin resistance index; MetS, metabolic syndrome; OR, odds ratio; CI, confidence interval

^1^stands for BPH, MetS or MetS-BPH

^2^stands for Non-BPH, Non-MetS or Non-MetS-BPH

^3^Student’s-t test

^4^Multivariate logistic regression analysis

The boldface represents statistical significance(*P* < 0.05)

## Discussion

The present study evaluated the relationship between polymorphisms of CYP17A1 in middle-aged and elderly men and multiple pleomorphic hormones, including T/E in patients with BPH, MetS, and MetS-BPH. It was found that only the genotype GG of rs743572, and the decrease of T/E were independently associated with BPH, MetS, and MetS-BPH. The increase in IR was in accord with the decrease in T/E, however, only MetS and MetS-BPH had independent contact with IR. So, it was speculated that the pathophysiological mechanisms of MetS-BPH are achieved by this gene’s regulation of T/E.

Previous studies have investigated whether MetS is related to lower urinary tract symptoms (LUTS) resulting from BPH [16] and the relationship between the SNPs (rs4646 and rs700518) of CYP19A1 and estrogen and MetS-BPH [15]. The current study went further to detect the middle-aged male crowd SNPs (rs743572, rs3781287, and rs4919686) of CYP17A1, and the research shows that genotypes GA and AA of rs743572, genotypes GG, GT, and TT of rs3781287, and genotypes CC, CA, and AA of rs4919686 were not associated with BPH, MetS, and MetS-BPH. Only the genotype GG of rs743572 was independently associated with BPH, MetS, and MetS-BPH after adjusting for age, TT, E2, T/E, INS, FPG, SHBG, DHT, IPSS, Qmax, PSA, TPV, and HOMA-IR. This finding provides a basis for us to plan a longitudinal study on individuals with the GG genotype of rs743572 to see whether individuals with this genotype will develop BPH or MetS or MetS-BPH. And, through such a study, it will be helpful to understand the temporal relationship and potential causal relationship between this genotype and BPH or MetS or MetS-BPH. In the meantime, if the results can be confirmed, it would be useful to detect the GG genotype rs743572 of CYP17A1 in clinical practice to predict BPH or MetS or MetS-BPH. At the same time, it also provides the possibility of precision medicine for these patients.

The current study further analyzed the relationship between related parameters of sex hormones and BPH, MetS, and MetS-BPH in the population distribution of genotype GG of rs743572. Findings show that only T/E had a common independent association with all BPH, MetS, and MetS-BPH patients. Compared with previous research [12, 13, 15–20], present results illustrate CYP17A1 gene SNPs in the Chinese population distribution, revealing that the genotype GG of rs743572 can be a common susceptible factor of BPH, MetS, and MetS-BPH. And, it was possible that this was done by lowering the T/E ratio. In this process, it was possible that IR plays an important role in MetS-BPH. Thus, we speculate that its possible mechanisms were shown in Supplementary Fig. 1, through CYP17A1 SNPs, especially through increasing the genotype GG of the rs743572 gene expression, decreased the activity of 17a hydroxylase, affected the conversion of progesterone into androgen, resulting in the decrease of serum testosterone and T/E ratio. This triggers IR, raises blood glucose levels, and promotes hyperplasia of prostate gland acinar cells and stromal cells in the prostate tissue, leading to the occurrence of clinical BPH and MetS. Moreover, through IR, MetS and BPH interact and progress clinically [16, 25, 26–30]. Again, combined with the previous literature, we speculated that small doses of aromatase inhibitor therapy, along with TRT, to adjust the balance of T/E ratios may have preventive and therapeutic effects on BPH and MetS in clinical procedures [11–16, 28, 29]. Of course, the proposed mechanism needs to be confirmed by further observation and research. And this is the next research topic to be confirmed for us. If this mechanism is confirmed, it will provide ideas for clinical treatment of different diseases, different treatments of the same disease, and precision medicine.

Overall, this retrospective cohort study has provided preliminary evidence of a potential biological link between BPH and MetS, which fills a gap in the field. This study has some limitations, and more comprehensive and methodologically robust research are needed to clarify these associations and their clinical implications.

## Conclusion

GG genotype of rs743572 in CYP17A1 gene can be an independent risk factor for MetS-BPH populations. And it probably works by reducing the activity of the enzyme 17a hydroxylase, affected the conversion of progesterone into androgen, resulting in the decrease of serum testosterone and T/E ratio, leading to MetS-BPH.

## Electronic supplementary material

Below is the link to the electronic supplementary material.

Supplementary Material 1: **Fig S1:** The schematic diagram for pathophysiological mechanism of benign prostatic hyperplasia combined with metabilic syndrome. SNP, single nucleotide polymorphism; BPH, benign prostatic hyperplasia; T, testosterone; E, estrogen; IR, insulin resistance; MetS, metabolic syndrome

Supplementary Material 2

## Declarations

### Registration

Registry used: Chinese Clinical Trial Registry. Unique Identifying number or registration ID: ChiCTR2200057632. Hyperlink to your specific registration: http://www.chictr.org.cn/showproj.aspx?proj=155496.
