# Supplementary material for: The GG genotype of rs743572 in CYP17A1 gene regulating the decrease of T/E ratio can be an independent risk factor for MetS-BPH: a retrospective cohort study
Source: World J Urol. 2024 Jul 24;42(1):439. doi: 10.1007/s00345-024-05138-3 (PMC11269469; doi:10.1007/s00345-024-05138-3)
Supplement: Supplementary file 2 — Supplementary Material 2 [file 345_2024_5138_MOESM2_ESM.pdf]

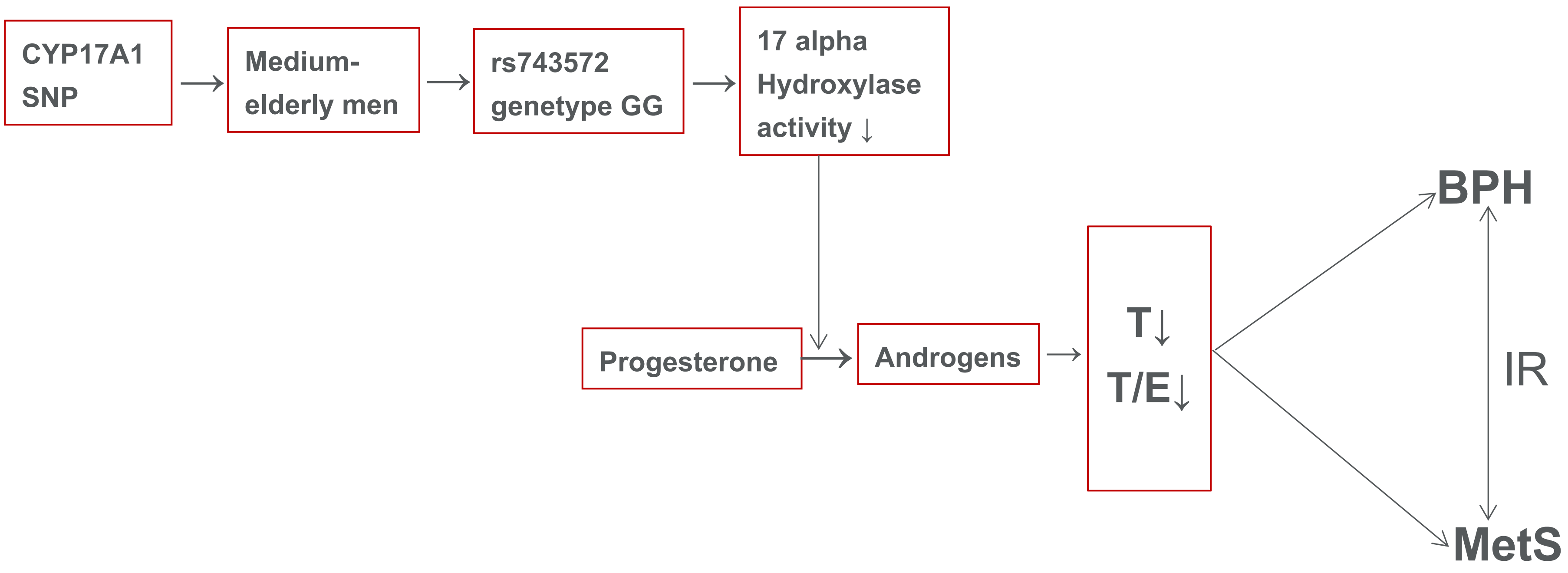

Figure 1 The schematic diagram for the possible pathophysiological mechanism of benign prostatic hyperplasia combined with metabilic syndrome. SNP, single nucleotide polymorphism; BPH, benign prostatichyperplasia; T, testosterone; E, estrogen; IR, insulin resistance; MetS, metabolic syndrome.
